# Supplementary material for: Association between Circulating Amino Acids and COVID-19 Severity
Source: Metabolites. 2023 Jan 29;13(2):201. doi: 10.3390/metabo13020201 (PMC9959167; doi:10.3390/metabo13020201)
Supplement: Supplementary file 1 [file metabolites-13-00201-s001.zip › metabolites-2065508-supplementary.pdf]

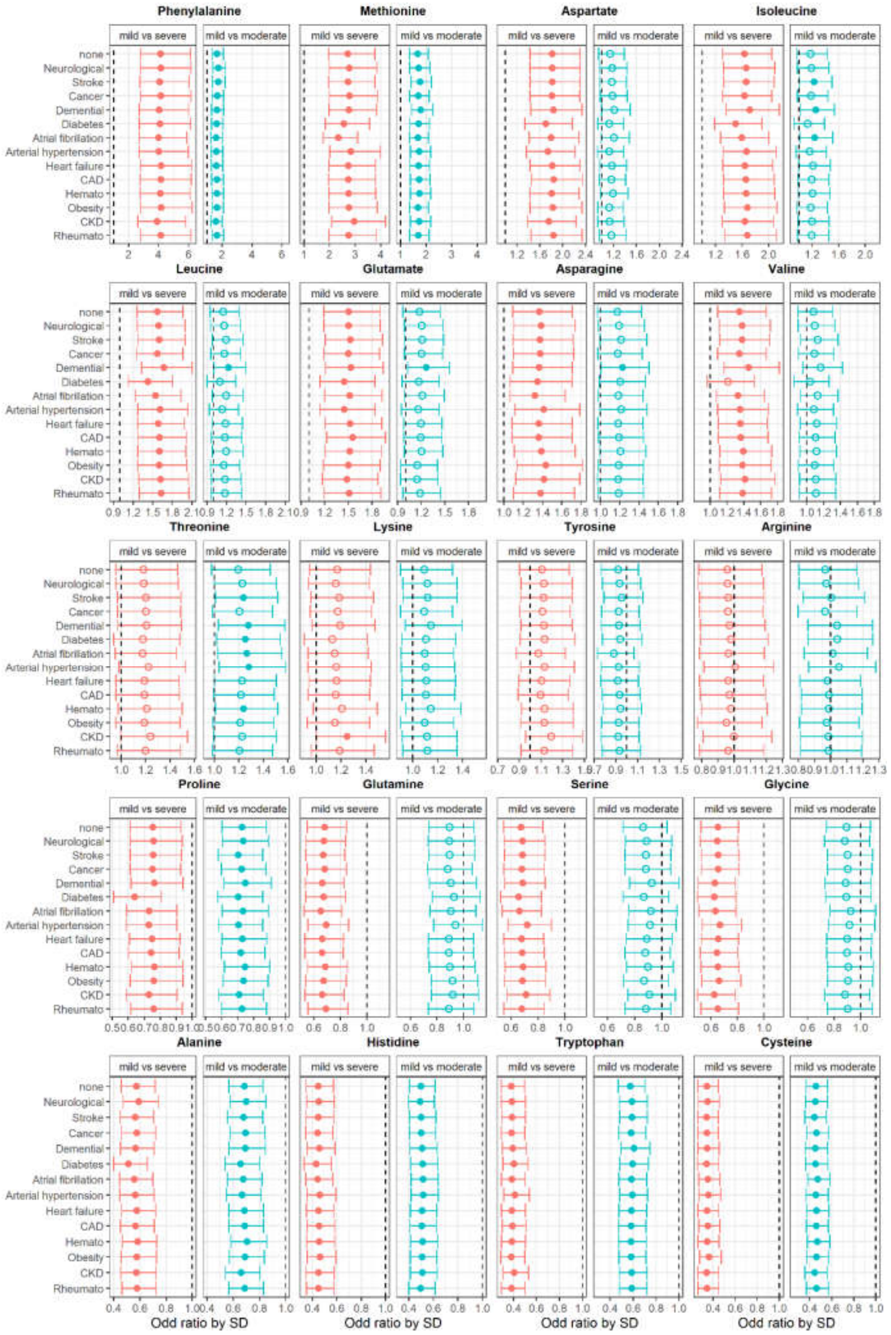

Supplemental Figure S1: Odds of having mild vs severe and mild vs moderate COVID-19 symptoms associated with circulating amino acids unadjusted and adjusted for comorbidities associated with disease severity. Results are presented as odds ratio per 1 standard deviation increase in amino acid. Models were either unadjusted (none) or adjusted for each of the comorbidities listed. The number of observations for each model varies, see Supplemental Table 1 for the number of observations available for each comorbidity. The x-axis scale is different for each amino acid. Full circles indicate a significant association and empty circles indicate a non-significant association. CAD: coronary artery disease, CKD: chronic kidney disease.

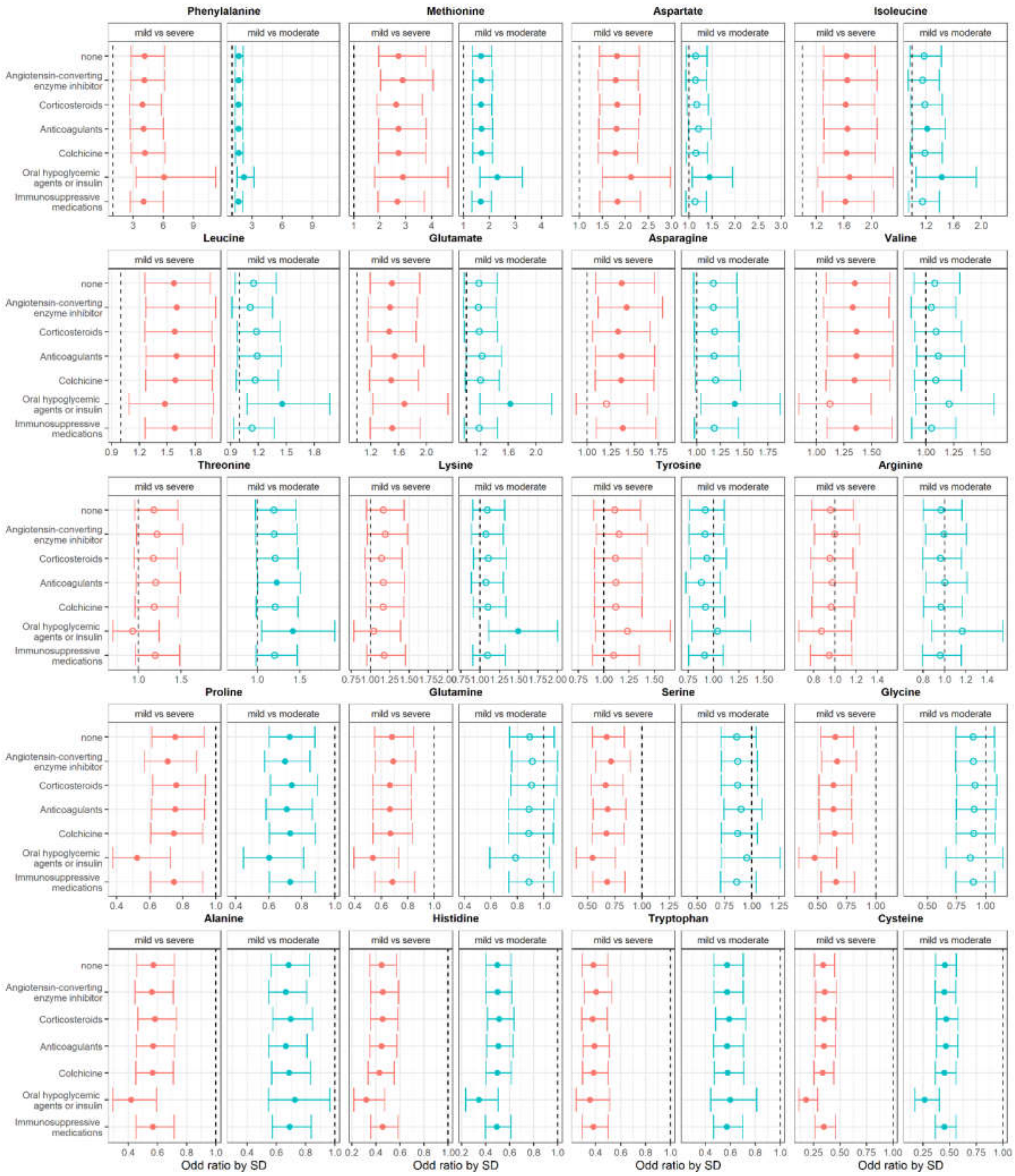

Supplemental Figure S2: Odds of having mild vs severe and mild vs moderate COVID-19 symptoms associated with circulating amino acids unadjusted and adjusted for medication intake at study inclusion. Results are presented as odds ratio per 1 standard deviation increase in amino acid. Models were either unadjusted (none) or adjusted for each of the medication listed. The number of observations for each models varies, see Supplemental Table 1 for the number of observations available for each medication. The x-axis scale is different for each amino acid. Full circles indicate a significant association and empty circles indicate a non-significant association.
